# Posthumous Extracts from the Veterinary Records of the Late John Field

**Published:** 1844-01-01

**Authors:** 


I. Posthumous Extracts from the Veterinary Records of the late *John Field*. Edited by his Brother, *William Field*, V.S. London, Octavo, pp. 236. Longman & Co.

II. The Veterinarian, or Monthly Journal of Veterinary Science. Edited by Messrs. *Youatt* and *Percivall*. London.

In our last number, (page 426,) we suggested the propriety of all medical men cultivating a more regular and extensive acquaintance with the various accessory branches of professional knowledge—branches which, although not indispensably necessary for the fulfilment of the ordinary duties of the Healing Art, cannot fail to have a more or less direct influence in establishing some of its most important general truths, and consequently in improving many of its practical details. Of these, Veterinary, or as it might be more properly called, Comparative Medicine is certainly one of the most valuable. The diseases of the lower animals are, in very many respects, quite the same as those which affect ourselves; they obey the same laws, arise from the same causes, and generally are remediable by the same or very similar means. The grand point of difference is the absence of mental or psychical influences in the one set of patients, and the often paramount and preponderating agency of these, in the other. In this respect, Infantile Medicine approaches nearer in its general principles and applications to the Veterinary Art, than the medicine of adults. The young child is exempt from the disturbing influences of those passions and emotions, which are apt at one moment to excite, and at another to depress the mental and corporeal energies of the grown-up man; so are all the lower animals. Hence it is that the diseases of both are, on the whole, of a simpler and less complicated character than those of adult human beings: more violent generally while they last, but certainly more under the control of remedies, and less disposed to be lingering and unmoved.

If indeed Veterinary Medicine had no other claim on the physician's attention than that of having suggested, and of having afforded the means of realising the truth of, *Jenner's* immortal discovery, this alone might be sufficient to demand for it his most respectful study. But there are other points—and those too not of trifling importance—in which the two branches of the Healing Art may be said to touch each other. The disease, for example, of Glanders in the horse, is unfortunately now known to be readily communicable to the human subject, exhibiting in him all its characteristic phenomena; and of late it has been alleged on good authority that the reverse also holds good; viz. that the matter taken from a glanderous human patient and inoculated upon a horse or ass will convey the genuine disease to them. Here then we have a remarkable instance of a mutual and alternating relationship.

Again, the history of the Malignant Pustule—to which we have adverted at some length in the Foreign Periscope of our last two Numbers—affords another proof of the intimate connexion between the diseases of the lower animals and of man. But there is a subject of still more extensive and important interest than either glanders or malignant pustule, and one that demands far greater attention than it has hitherto met with from our profession—we mean the relations that exist between the origin and diffusion of the Epidemic diseases of man on the one hand, and of the Epizootic diseases of horses and cattle on the other. Much curious and most valuable information remains to be discovered on this interesting topic of professional research—information that may yet lead to some precious improvement in all that pertains to the hygienic and sanatory discipline of every country without exception. Our neighbours, the French have on this, as indeed on most other subjects of professional research, anticipated our exertions. Let us follow their good example without delay, and strive to come up with, if not to get the start of, our *devanciers* in this very useful pursuit. We mentioned, some time ago, that M. *Rayer* had begun a new Journal on Comparative Medicine; and already several numbers of this periodical have issued from the press. This fact alone shews the importance which that distinguished physician attaches to this hitherto unexplored—at least by physicians—field of professional enquiry. To the credit of France also let it be told, that, for some years past, its far-famed Institute has so thoroughly recognised the importance of Veterinary Medicine and the respect that is due to its professors, as to have elected, on a recent occasion, M. *Barthelemy* to the dignified post of the president's chair. The Royal School at Alfort, near Paris, is a noble establishment, and is often the daily resort of some of the most eminent physiologists and practitioners of that metropolis. Hitherto the military veterinary surgeons have not occupied the position in the French army, to which they are, if men of education, most justly entitled. They rank only as *sous-officiers*, not much above the master-tailors and master-saddlers of the regiment. But from the well-known impartiality and active sagacity of the present illustrious Minister of War, it is confidently expected that this evil will very soon be rectified. If there was no other motive to prompt the change, that of the national interest and welfare will inevitably force any ministry to attend to it without much delay. The expense, to which France is annually subjected for the maintenance of her Cavalry Establishments, is enormous; and it is said that much of the present outlay might be avoided, if a greater encouragement was held out to expert and well-instructed veterinary surgeons attaching themselves to each corps.

The following paragraph from a well-written appeal of M. *Renault*, the Director of the Royal Veterinary College at Alfort, to the French Parliament, on the claims of his professional brethren, will be read with interest.

" If there is a fact," says he, " incontestable and unfortunate, it is the frequency of the diseases which prevail among the horses of our regiments, and the frightful mortality by which they are decimated. Every year our losses of this kind, and at the same time the millions of money which they subtract from our treasury, compromise very seriously our military situation, by weakening our cavalry force........We, more than any other nation in Europe, should feel a deep interest in the preservation of our cavalry, because we, more than any other country, have not the means of replacing them." M. *Renault's* energetic remonstrance will have its effect. As we have said above, the motive of self-interest will, ere long, force nations, as it does individuals on all occasions, to grant what perhaps might not be conceded to the claims of justice.

It would seem, from an interesting paper by Mr. *Wilde* in the July Number of the Veterinarian, that the Austrian Government is fully alive to the importance of having an efficient corps of veterinary surgeons, not only attached to their army, but also dispersed over the country. The Veterinary Institution at Vienna is most liberally endowed, and very admirably conducted. The course of study prescribed is certainly, as far as we can judge from the report, very complete—embracing not only the Anatomy and Diseases of the Horse and other domestic Animals, but also Natural History and Hygiene, Chemistry and Physics, and special lectures on Epidemics, Epizootics, and Veterinary Police. In Austria, many of the general medical practitioners and surgeons have attended the lectures, and graduated at the Veterinary College. Those gentlemen, being afterwards distributed over different parts of the Empire, practise both branches of the profession, with no inconsiderable advantage, both to themselves and to the inhabitants. Would not such a custom as this, we may fairly ask, be much more creditable among ourselves, than the too frequent one of country apothecaries, or *doctors* as they are usually called, eking out their incomes by selling tea and sugar, perfumery, and cigars? Let no medical man suppose that there is any thing derogatory in following an occupation which such men as *Coleman*, *Blaine*, and *Field* have adorned, and in which Messrs. *Youatt* and *Percivall* are now engaged.

To these two last-named gentlemen, Veterinary Medicine in this country is deeply indebted for their successful labours in elevating it as a liberal calling. Their excellent works are known to many out of their profession, and every one in it, we believe, is willing to admit their great practical skill. The numerous writings of Mr. *Youatt*, in particular, have exercised a most salutary effect, in drawing general attention to his favourite pursuit. His enlightened zeal in promoting, by every means, a more liberal course of education among the veterinary students and younger members, and in uniformly inculcating the precepts of humanity and benevolence, cannot fail to have a truly beneficial influence upon all.

" I would urge," says he, " upon the young pupil to make himself acquainted with the chemistry of agriculture, the history of the growth and characters of plants, and the different processes by which they are brought to maturity. I would have him study the history and character, as well as the management of the different breeds of cattle—having also in reserve the anatomy, and diseases and management of all our domestic animals. I would have him be perfectly acquainted with these things, and he would gradually regain the esteem and confidence of the agriculturist, and be his chosen friend and adviser."

The impulse, that has of late years been given to the study of agricultural chemistry, and the growing enlightenment that bids fair to spread among our farming population generally, must be felt, and we doubt not, will be duly appreciated by those, who profess to be acquainted with the habits and diseases of the lower animals. Thus the improvement of one class will inevitably induce that of another, with which it is brought into contact, and the general result will be a wider diffusion of useful knowledge, and an elevation of all sorts and conditions of men to a higher position in the social scale.

It will afford us great pleasure to contribute to this good cause by an occasional article on Veterinary Medicine in this Journal; the present may be taken as a pledge of our good wishes and intentions.

The Posthumous Extracts from the papers of the late Mr. *John Field* may be described as Clinical—or shall we call them, Constabular ?—Reports of Diseases among Horses. It is a work of very curious and instructive information. The "Veterinarian" is a well-established journal, under the joint superintendance of Messrs. *Youatt* and *Percivall:* it has already attained its XVI. Vol. The numbers, to which we may make reference in the subsequent observations, are those from January to October of last year.

The first subject we select for comment is that of Glanders and Farcy. Several cases are related by Mr. *Field;* but, before alluding to these, we shall make a few general remarks on the leading features of these allied diseases.

Till within the last eight or ten years, it was generally believed that they belonged exclusively to the horse, the ass, and the mule. To these we must now add the human subject; not a few cases having been observed in men who have had to do with glandered horses, both in this country and on the continent—the disease having been transmitted either by the direct contact or inoculation with glanderous matter, or by the atmosphere becoming tainted and infected.

We have said that Glanders and Farcy are allied diseases. Either of them may exist alone and by itself; or they may be co-existent one with the other. Hence veterinary writers talk of Simple Glanders, and of Farcy-Glanders. Of both these diseases there are two kinds or forms—the acute and the chronic.

Let us first attend to Acute Simple Glanders.

Its characteristic symptoms in the horse are described to be—intense inflammation of the pituitary membrane, attended by erosions, which soon pass into chancre-like sores—swelling of the lips and nose—rapid extension of the ulceration, giving rise to a purulent nasal discharge, which often becomes a purplish or bloody fœtid sanies—subsequently gangrene of the Schneiderian membrane, occasionally with hæmorrhage—painful swelling of the sublingual glands—inflammation of the conjunctivæ and eyelids, quickly passing into a livid and swollen state, with an offensive discharge, and—fever of a putrid or malignant character. The breathing becomes more and more hurried and oppressed, and the superficial vessels congested with blood. The animal usually dies within a few days, if the disease does not pass on to the second or chronic stage.

When the foregoing state is complicated with Farcy, the disease, as we have said, is called Farcy-Glanders, and then it exhibits the following additional phenomena, besides those already mentioned—tumors about the legs, lips, face, neck and other parts of the body, which are at first hard, and afterwards soften, burst, and degenerate into foul ulcers.

These tumors vary a good deal in size in different cases. In some cases they are small, and create little distress; while, in others, they are exceedingly large and painful, and rapid in their course. Numerous lines of communication are usually observed between these tumors or ulcers, more especially when they are situated on the inside of the limbs: these lines are caused by inflamed absorbent vessels.

The writer, from whom we have borrowed these remarks, propounds the following pathological views, in reference to the two diseases. "We see clearly," says he, "that Glanders is essentially a disease of the respiratory organs, although other parts become implicated during its progress. We see also that Farcy is an affection of the glands and absorbents; and that each exhibits the same, or nearly the same, symptoms, and pursues the same course in the human subject as it does in the horse. Although these varieties of disease are developed in different structures, and although it may not be easy to give a satisfactory explanation of the nature of their relationship, still their identity is a fact, which experimental enquiries have fully established. The Glanders may appear first, and the Farcy be superinduced; or *vice versâ*. But recent investigation has gone further than this, and has clearly proved these two facts; *first*, that, with the matter taken from the nostrils of a glandered horse that is quite free from Farcy, we can induce this latter disease, and often too in its simple uncomplicated form, in another animal that is perfectly healthy; and *secondly*, that simple Glanders may be generated merely by inoculation with the matter of a simple farcied tumor. This is not indeed what we should have expected, reasoning from analogy. We might naturally suppose that the matter, that is inoculated, would produce the exact type of that form of the disease by which it was generated, and not one of a different aspect, and belonging to a different structure. In Glanders, it is the mucous membrane of the respiratory organs that is essentially affected; while, in Farcy, the absorbent vessels and glands are unquestionably the seat of the morbid action. The considerations, to which we have now alluded, are of great importance, as having a direct bearing on the treatment of *Glanders*, which our present knowledge unfortunately does not enable us to explain with sufficient precision."

The remarks, which Mr. *Field* makes on these two important diseases, are, it is to be regretted, very brief and unsatisfactory.

"Farcy," says he, "is a disease of the superficial absorbents; they become inflamed. It is produced by variation in temperature, or by unequal exercise. It attacks post-horses particularly. It seldom arises from contagion; for by inoculation glanders is produced. Glanders is also a cause of farcy. We should treat it at first as an inflamed part; subsequently by blistering with the infusum lyttæ, or by firing, by which it is frequently resolved." 176.

The most interesting fact, connected with the cases recorded by Mr. *Field*, appears to us to be, that in almost every one—they were of the chronic form—the lungs were found on dissection to be more or less deeply affected with tuberculous disease. One case, indeed, is headed, "Pulmonary Consumption with appearances of Farcy." The impression certainly that has been left on our minds, after the perusal of the various data which we have found in the "Posthumous Extracts," and the Veterinarian, is, that the (chronic at least) forms of Glanders and Farcy are, in very many respects, analogous with scrofulous disease. Unquestionably there is a something superadded to the morbid condition or cachexy, to which the term Scrofula is given; and that *something* is no less than the existence of a virulent and contagious poison, which is capable of direct transmission from one animal to another. Nevertheless, there are not a few points of resemblance between these two equine diseases on the one hand, and that of Scrofula in the human subject on the other; and not the least remarkable of these is the frequent tuberculated condition of the lungs in fatal cases of the former. A statement, in one of the late Reports of the Veterinary School at Alfort, seems to afford some confirmation of this pathological view. It appears that, among the first and most frequent signs of the Chronic Glanders observed at that Institution during the past year, was an engorgement or inflammatory enlargement of the testicles, which usually terminated in a suppurative disorganisation of these organs.

This disease seems to have exhibited not a few of the characters of strumous degeneration of tissue. At least, this idea suggested itself to our minds, when we were reading the description given of it in the Veterinarian.

The same Report proceeds to inform us that, during last season, the Acute Glanders prevailed to an unusual extent among the horses in and around Paris, and more especially among those that were engaged in the toilsome works, connected with the building of the fortifications in the neighbourhood of that metropolis. The excessive fatigue, to which the horses were subjected, is supposed to have been the cause of the great prevalence of the disease; many of the poor animals being often kept at work for 18 out of the 24 hours. As a matter of course, the disease, when developed in one animal, had a tendency to diffuse itself by contagion to others near it. Fortunately, however, the epidemic, although wide-spread, was not of a very malignant character; the mortality being not at all comparable to that, which occurred two years ago. Moreover, there was scarcely a single instance of the disease having been communicated to a human being during the past season.

Some interesting facts, relative to the transmission of Glanders, are next mentioned in this French report. For example, it appears from certain experiments that the activity of the virus is not at all mitigated by repeated inoculations; but that this, like the genuine cow-pock matter, retains all its contagious properties as powerfully then, as when first taken from its original source. The virus has been repeatedly inoculated on cows, dogs, sheep and rabbits ; but, on no occasion, has the genuine disease been ever developed in any of these animals ; whereas, the same matter inserted into a healthy horse or ass has almost invariably induced the disease.

"We have also tried," continues the reporter, "whether the matter of the nasal discharge, when dried in the open air, preserves its virulent property for a considerable length of time ; and we have seen the eschars (?) proceeding from this maceration, macerated and distilled in water, and yet the part being inoculated at the end of six weeks, acute Farcy has appeared. We have tried whether the virulent matter existed elsewhere as well as in the nasal secretion, and we have seen matter from ulcers of the lungs very rapidly producing acute Glanders. The blood too possesses the same virulent properties. Injected immediately after its extraction from the vein of a diseased into the circulation of a sound horse, it produced, at the end of four or five days, a Glanderous eruption."

Glanders, it is well known, is quite the *opprobrium* of the veterinary art ; and unfortunately there seems but little prospect of a successful remedy, or plan of treatment, being discovered. Hear what an able member of the profession in France says on the subject :

"In this year, as in all the preceding ones, we are compelled to confess the complete impotency of all our efforts to cure chronic Glanders. This statement will astonish no one, who is aware of the irreparable lesions which the disease leaves behind. Whoever has witnessed the profound destruction of the Schneiderian membrane—the collections of purulent matter in cavities almost closed—the complete transformation of the pellicular membrane which once closed the walls of these cavities—and if he has examined the lungs and seen the large masses of compact tuberculous matter deposited in their substance, and at the same time observed the serious lesions in different parts of the absorbent system, he will not be much surprised at the impotence of all remedial measures."

The following instance of successful treatment in a case of Farcy-Glanders is related in one of the numbers of the Veterinarian.

A six-year old horse was observed to have a dull drowsy look—his head thrust out, his nose, windpipe and chest being nearly horizontal—and his breathing laborious, from the general swelling of the parts about the throat. He made a noise at every inspiration. On looking into the nostrils, they were found incrusted with purulent matter, and a greenish-yellow discharge flowed out. The Schneiderian membrane was of a dark livid hue, with petechial patches here and there. The submaxillary glands were enlarged and indurated. The fore and hind legs were much swelled and spotted over with pimples—evidently absorbent glands. Some would have called them Farcy-buds, although they were not so deeply-seated as these generally are : a thin ichorous discharge oozed out from many of them.

The horse, having previously been removed to an airy box, was bled to the amount of nine quarts : the blood, on coagulating, exhibited a great deal of the buffy coat. Several of the tumors on the legs were freely laid open ; an aloetic ball given, and a blister applied to the throat. The nostrils were ordered to be frequently washed out with a solution of the chloride of Lime. Next day, he was again bled to five quarts, and the purging ball repeated : digestive ointment was applied to the opened abscesses. Although many more abscesses—some of a large size—

formed on different parts of the body, and the horse became exceedingly weakened and emaciated, a cure was ultimately effected, "although," says the narrator, "he bears fearful scars on the many parts of his body, where suppuration had taken place."

We may here with advantage introduce two or three abridged reports of cases, that have been recently observed in the human subject, and are narrated in the communication of "Erinensis" to the Veterinarian.

*Case* 1.—A waggoner, 19 years of age, was admitted into the Hôpital de la Charité on the 18th of October. After a week's general *malaise*, he was seized with intense pains in the ankle and knee-joints and in the muscles of the leg and thigh. The pulse was quick; the thirst great, and fever high. On the 25th, several pustules appeared on the instep and upper surface of the toes of the left foot. They burst, and afterwards healed up in the course of a few days; but then a diffused swelling appeared on the front of the upper third of the thigh, followed by two similar swellings, one on each leg. For eight months, a succession of tumors of a similar kind appeared on almost every part of the upper and lower extremities. With the exception however of weakness and emaciation, there was nothing in the state of the patient's health to excite alarm. He was treated, throughout the whole of his illness, with bark and wine. On the 5th of July in the following year, (from this we may judge of the tediousness of the disease), Iodine, with the hydriodate of potash, was administered. Soon after this date, he was discharged quite cured.

*Case* 2.—A groom, who slept in a stable occupied by a glandered horse, was attacked, some days after the death of the animal, with the same disease—characterised by the eruption of pustular and gangrenous sores over the body, in the nose and throat, beneath the ears, on the glans penis and on the feet. The evening of his death, a small quantity of matter was collected on watch-glasses from the gangrenous sores beneath the ear, on the fore-arm, back and shoulder. This was afterwards inoculated upon a foundered mare, which gradually became affected with all the genuine symptoms of Glanders and Farcy. The result of this experiment shews the nature of the disease, of which the groom died. The horse usually catches the disease by the way of atmospheric infection, and not by the direct inoculation of the morbid matter. There is every reason to believe that the human patient also caught it in the first of these ways.

The following is narrated as an instance of severe *Chronic Farcy* in the human subject.

"M. *Leone*, veterinary surgeon in the French army, opened a farcied abscess in a horse belonging to his regiment. As the abscess was large, he introduced his hand into its cavity, to ascertain its extent. There happened to be at the time a slight abrasion on one of his fingers. In the course of a few days this spot became painful, enlarged in size, and covered with fungus-like growths. The wound was cauterised; but it did not heal for three months. Besides the local affection, several painful hard tumors, like those of farcy-buds, made their appearance on the inside of the left elbow. The joint itself became swollen and painful. An abscess formed and was opened. Others succeeded, and fistulous sores were established. Subsequently the right knee-joint, and afterwards the instep and foot, became similarly affected. Tumors formed, and soon broke."

Before dismissing the subject of Glanders and Farcy, we would earnestly recommend to those, who have the opportunities of personal observation, to examine with care the physical and microscopic appearances of the blood,* as well as of the purulent discharges; and to try the effects of some of those saline and mineral medicines, which are known to exercise a marked influence on the condition of the circulating fluids—such as common salt, the chlorate and nitrate of potash, &c., and the preparations of mercury and steel.

We should now, if space permitted, proceed to examine at some length the history of another Epizootic that is, as we have already said, of great frequency and danger among horses, cattle, and other animals—to wit, the disease, or perhaps we should rather say the variety of diseases, to which the term of *Influenza* is given.

Mr. *Field* thus describes the symptoms of the Epizootic of 1819:—Quick full pulse; moist tongue, but feverish smell and red appearance; eyes much inflamed in some cases, in others extremely dull and heavy; and eye-lids closed, indicating severe pain in the head. The breathing not much quickened or oppressed, unless just previous to dissolution. Blood drawn with great difficulty, and, when coagulated, extremely firm and shewing a thick buffy coat. In some cases, tremendous swellings in different parts of the body, especially of the legs.

The disease of 1823 was still more severe and fatal. It affected the whole body with such excessive inflammatory action in all its parts and organs, that the animal was very quickly reduced and destroyed by it. The most active antiphlogistic treatment was necessary; those cases doing best, where the regime was most vigorous, provided purging was not excited; for, when that was severe, the animal generally died.

But let it not be supposed that Influenza among the lower animals is always, or even generally, of so very inflammatory a type, as represented by Mr. *Field* to have been the case during the Epizootics of 1819 and 1823. The danger indeed generally arises from the *pleuro-pneumonia* that may be present; but then there is usually existing at the same time such a prostration of all the vital forces as to indicate an adynamic, if not a typhoid, state of the system. The signs and symptoms in unfavourable cases are reported to be—great difficulty of breathing, the expiration being accompanied with a grunt—absence of the respiratory murmur over various parts of the chest—bronchial respiration round the margin of the hepatised portions—increasing prostration of strength—quick but feeble pulse—decrease of animal heat—torpidity of the nervous system—dysentery or diarrhœa—and an accompanying fever of a low typhous character.

"The characteristic pathological feature" says a most intelligent writer in the Veterinarian, "of this disease is an early effusion of lymph into the pulmonary vesicular structure and bronchial tubes, as the termination or natural means of

---

* We may here observe that it would be well if veterinary writers were more in the habit of recording in all cases the state of the blood, when drawn and allowed to coagulate. Unless this is done, it is not possible for the reader to form any accurate conclusion as to the propriety, or not, of the practice in any particular case. Our *confreres* must have observed that this is invariably attended to, in drawing up clinical reports.

relieving inflammation in their parietes and parenchymatous tissue generally. There is also an effusion of water and lymph into the thoracic cavities. Except the bronchial glands being occasionally in a state of suppuration, I have very seldom found any appearance of pus in this disease."

He then cautions his readers not to allow themselves to be led into the very dangerous error of regarding the disease as mere ordinary bronchitis, pneumonia or pleurisy, and such as to require the usual active depletory remedies. Antiphlogistic measures may be necessary in the very early stage and to a certain extent; but their effects must be very carefully watched, and they should never be pushed very far. Calomel and the hydriodate of potash have been found of essential benefit, especially when they are combined with nitre, opium, and the tartrate of antimony. Blisters also should be applied along the course of the trachea and over the sides, from the spine to the sternum.

We have omitted to mention that the heart and its capsule are often very seriously affected in this disease. Indeed one gentleman seems to suppose that it essentially consists in a subacute inflammation of the central organ of the circulation,—this being accompanied however with symptoms of a typhoid state of system, and often requiring the use of diffusible stimulants internally, and blisters outwardly.

*Pleurisy* is a frequent disease among horses. It may be either acute or chronic. The premonitory symptoms are trembling or shivering, and a *staring* of the coat, (this, we suppose, is equivalent to the *Cutis anserina* in the human being). When decided, the disease is easily recognised; the pulse is full, hard, and wiry; the breathing is uneasy and restrained, the inspiration being quick and interrupted, while the expiration is slow and prolonged; the animal is stiff and unwilling to move; he stands with his neck extended out and his nose protruded; pressure on the side gives pain, causing the animal to *grunt*, followed by convulsive twitchings of the Panniculus Carnosus. Similar twitchings succeed a faint suppressed cough, or rather sneeze, causing a trembling folded appearance of the integuments, and lasting some seconds.

In not a few cases, the inflammation of the pleura terminates in effusion into the chest—indicated by abatement of the fever symptoms, increased dyspnœa, anasarca of the legs and integuments of the chest and abdomen, dulness on percussion, and indistinct respiratory murmur in the inferior costal regions.

The last stage of the disease is marked by the following symptoms: the legs are extended and separated; all the auxiliary respiratory muscles are called into action; the dilated nostrils, staring eyes, anxious countenance, and laborious heaving, strikingly indicate the threatened suffocation. A mucous rattle is heard in the windpipe, the extremities become cold, the pulse fluttering or scarcely to be felt, and death soon closes the scene.

As in the human subject, chronic or rather subacute Pleurisy is apt to come on in a very insidious manner; and hence the disease is often far advanced, before application is made to the Veterinary Surgeon. We need scarcely say that the substance of the lungs is, in many cases, implicated at the same time; then, we have a case of genuine Pleuro-pneumonia; the *post-mortem* appearances of which, in the horse, are exactly those observed in man. A bad form of the disease is that often met with during the prevalence of epizootic febrile disorders, or *influenzas*, as they are often called.

The cause of the increased danger is that there is a typhoid or adynamic state of the system at the time. A similar remark is applicable to that form of pulmonic inflammation, which not unfrequently supervenes after severe or extensive burns.

Pneumonia in the horse is occasionally followed by the formation of vomicæ or abscesses in the substance of the lungs: these are sometimes quite black and gangrenous. In such cases, the expired air is most fætid and offensive.

Mr. *Field* relates a case of Laryngitis or Croup, threatening suffocation, in which tracheotomy was performed with success. It was necessary to keep the wound open for a considerable time, as the aperture of the glottis seemed to have become much narrowed.

We now pass on to notice a very remarkable form of Hepatic Disease in the horse, which well deserves the notice of the human pathologist.*

It appears that, though a horse seems to be in perfect health, and is moreover capable of laborious work, the liver may have become so softened and pulpy and its peritoneal covering so loosely adherent to its parenchyma, that a sudden exertion will cause a rupture of the viscus, and an immense extravasation of blood into the abdomen, to take place. The accident, as a matter of course, is generally fatal, and the death is sudden. The curious circumstance in this disease is that, in most instances, there are no premonitory symptoms of any hepatic derangement; the animal often has been remarkably healthy, the biliary secretion regular, and the alvine excretions natural. When the extravasation is large and rapid, the symptoms are those of internal hæmorrhage—pulse irregular and indistinct; action of heart tumultuous; profuse sweating; pawing; curling of the upper lip and pouting of the nose; lifting the hind legs to the belly; hurried respiration and deep and frequent sighing; distended abdomen; blanched conjunctiva and buccal membrane; amaurosis; extreme debility; fainting and death.

In some cases, however, although the parenchyma of the liver has degenerated into one huge mass of bloody pulp, its peritoneal coat remains entire, or is only slightly ruptured. The symptoms then are much more indistinct than those we have just enumerated, and often far from being satisfactory, as regards the diagnosis. Weakness and faintness at work, diminished appetite, swelling of the hind legs, fullness of the abdomen, buccal and Schneiderian membranes pale, pulse frequent and feeble, occasional sighing, partial amaurosis, with dilated pupil of one or both eyes—these are the most common features of the case.

Mr. *Field* mentions a curious circumstance, connected with the lesion of the sight.

---

* "The disease is called by Mr. *Field,* and other veterinary writers, Hepatirrhœa. Would not Hepatorrhagia be more correct? The one word expresses a *flowing*, the other a *rupture* or *rushing out*; the Greek roots being ρεω in the one case, and ρηγνυω in the other.

"It is a remarkable fact," says he, "that, notwithstanding the patient may rally for a time from the disease, and regain his condition and strength, and return to work, I have neither seen nor heard of a single instance of the recovery of the sight, although in one case the horse worked for twelve months afterwards."

When the case has proved suddenly fatal, the abdomen is found on dissection deluged with blood, sometimes to the amount of ten gallons, or upwards. The peritoneal coat of the liver is usually found to be rent in one or more places, and its parenchymatous substance reduced to a soft lacerable mass, in some places so broken down that small portions of solid matter float in the semi-fluid or grumous mass into which other parts are converted. The weight of the liver and coagula has been known to exceed sixty pounds.*

An interesting case of Hepatorrhagia is published in the January number of the Veterinarian. A fine fiery horse—that had been hunted the day before with some harriers, and had been ridden (in the true spirit of larking,) over several rasping fences, which he had done in an extraordinary manner—was suddenly taken ill. He had seemed in perfect health up to the time of seizure. When visited, he was apparently in considerable pain, with distended nostrils, laborious breathing, and rapid tumultuous action of the heart.

He died very shortly afterwards. On dissection, the liver was found excessively diseased; not a fifth part of its substance remained; the rest had become absorbed; and the peritoneal covering was distended to an enormous extent with coagulated blood. It had given way at one point, and allowed the escape of the blood into the abdominal cavity.

The violent exertions, which the horse is so often made to undergo—taken in connexion with the circumstance of the absence of a gall-bladder, and the consequent liability of the biliary tubes to extreme distention on many occasions—seem to be the main cause of the disease in question.

The liver in the horse is also liable to the formation of Abscesses in it,—either single and large, or numerous and of smaller size, being dispersed through its parenchymatous tissue. In one case, no less than 29lbs. of pus were found in one hepatic abscess.—At other times, the liver is affected with general enlargement or Hypertrophe.—A curious case is related of Melanosis involving all the abdominal viscera, the liver among the number: the spleen was of an enormous size and weighed 67lbs.; its natural weight not exceeding four or five pounds.

We shall now glance at some of the diseases of the Intestines.

The following vivid enumeration of the symptoms, which accompany Ileus from intus-susception of the bowels or any other cause, is interesting.

"Pain, restlessness, in some cases approaching to madness; unrestrainable; wandering about; rolling on the back; sweating, in some cases profuse; crouching; sitting on the hind quarters, almost diagnostic; anxious countenance;

---

* The hæmorrhage, Mr. *Field* says, is from the branches of the *Vena Portæ;* for, if water be injected into this vessel, the *Vena Cava posterior* having been previously tied, it will escape from innumerable orifices in the abraded parts of the liver.

frequent feeble pulse; belly at first of natural size, subsequently fuller, in some cases distended, dependent upon the locality of the intus-susception; membranes, in advanced stage, turgid, injected; mouth moist and clean, or furred and offensive; respiration accelerated; continued restlessness; rearing with fore legs into manger, and standing upon that *point d'appui;* looking back from side to side; extremities cold; pain absent, tranquil; sighing or snorting; death. The sighing may exist in some cases, and not in others; and in some, retching and vomiting at stomach."

The picture is quite admirable from its truthful force. The most frequent seat of Intus-susception in the horse is where the ileum joins the colon. On being called to such a case, the veterinary surgeon is very properly enjoined by Mr. *Field* to "examine the rectum, the groin and abdominal rings, and the neighbourhood of the umbilicus for hernia." The advice should not be forgotten by the physician: cases are every now and then occurring of very awkward mistakes being committed by not attending to it.

Cold applications, clysters of oak-bark, and injections of vinegar and water are recommended. Why not rather tobacco enemata, with or without bleeding? Our author makes no mention of purgative medicines; are we to infer that he does not approve of them? Would that they were less used in cases of obstructed bowels occurring in the human subject! often they do infinite mischief, and only aggravate the existing impediment.

*Hernia* appears to be a not unfrequent complaint in the horse. Several cases of Scrotal hernia are related by Mr. *Field.* Our readers may like to know how the protrusion is reduced. Here is one of the clinical reports.—A horse was brought to the hospital, suffering much pain, which had lasted for more than six hours. The off-side of the scrotum was full and tense, but not crepitant; the spermatic cord more than doubled in size. The animal was immediately cast and laid upon its back. My right hand, introduced *per rectum,* readily grasped the intestine, which was in front of the spermatic cord; and by carefully drawing it downwards, an assistant at the same time elongating and compressing the scrotum and drawing it upwards, the knuckle of intestine at first gradually, and then suddenly, slipped in. He was afterwards bled, and three days afterwards discharged "perfectly cured."

In one of the cases reported, an operation was necessary, the *taxis* failing of success, and the intestine being strangulated.

"An incision was made through the integuments at the upper part of the scrotum into a mass of congealed extravasated blood, surrounding the testicle, at the upper part of which were two convolutions of intestine through the abdominal ring: they were not greatly inflamed. Having enlarged the ring by means of the bistouri cachée, the intestine was returned. The horse shewed no symptoms of pain in the belly. At night, the testis on that side, being in a gangrenous state, was removed, a ligature being placed on the artery." 99.

The patient died on the second day afterwards. On dissection, a portion of the ileum, which had been strangulated, was found in a sloughy state.

An instance is mentioned, where the hernial swelling was mistaken for an abscess, and an opening made into it with a lancet; and another, where a strangulated Ventral Hernia was completely hid by the penis: the animal died unrelieved.

As in the human being, a portion of intestine sometimes passes into or through an opening in the diaphragm, giving rise to all the symptoms of strangulated Hernia, while there is no external swelling at any part of the abdomen. Sometimes the symptoms are far from being strongly marked during life; and then there is necessarily a good deal of ambiguity in the diagnosis. In one case of this kind, where the animal died on the second day, the contents of the abdomen, on dissection, appeared at first to be quite natural; but, on examining more carefully, it was found that several feet of the Ileum and Jejunum had passed through an opening in the tendinous part of the diaphragm on the right side, and were strangulated. Mr. *Field* very wisely concludes his report by remarking, "how necessary it is to be exceedingly cautious in cases of this description, not only in speaking of the result, but also in forming too hasty an opinion as to the seat of the disease."

Several cases of *Rupture of the Stomach* are related; two also of ruptured Intestine—in the one case the colon, in the other the rectum, was the affected gut. We have likewise an instance of sudden death from Hæmorrhage into the stomach, and another from the bursting of the mesenteric vessels, and the consequent extravasation of blood into the cavity of the abdomen.

The formation and impaction of Balls and Concretions in the intestines are often the cause of alarming illness in the horse.

Among the abdominal cases is one, which is headed "Paralysis of the Intestines." A horse was bled and opening medicine given, in consequence of a swelling of his legs, &c. Two days afterwards there were symptoms of colic, for which some anodyne medicine was administered. The pain continuing next day, the dose was repeated twice. On the following day, there was considerable restlessness and tension of the abdomen; no evacuation. Four pounds of blood were taken; this caused great faintness. Clysters were rejected as soon as administered; and the last attempt to administer one of spirits of turpentine and linseed oil produced immediate fainting; and in two hours afterwards the animal died. On dissection, the stomach and colon were found to be much distended with fæces and confined air, but there was not the least appearance of inflammation in any of the abdominal or thoracic viscera. The enormous swelling of the belly and other symptoms sufficiently account for the death, which (says Mr. *Field*) was caused by Paralysis of the Intestines.

In another fatal case, in which there had been constant vomiting for some time before death, the duodenum was found much distended for about a foot and a half of its length, and then exceedingly contracted, as if it had been tied with a band. On opening the intestine, it was found to be extremely inflamed, almost verging to gangrene, just anteriorly to the pallid and contracted portion. These cases will naturally recal to the reader's mind some of the views of Dr. *Abercrombie*, relative to intestinal disorders.

*Tetanus* appears to be most frequently induced in the horse by some injury or irritation of the foot; such as the puncture of a nail, or sharp piece of bone, a deep-seated abscess, &c. Sometimes it follows the operation of docking the tail. The usual symptoms are—retraction of the eyes within their sockets; the membrana nictitans more or less protruded; the ears stiffened; the jaws closed and perhaps firmly locked; the nostrils dilated; the abdominal muscles much contracted; the back more or less rigid; the tail elevated and tremulous; the limbs stiffened and extended, and every now and then tremulous with spasm. In one case, we read that there was "dreadful Opisthotonos, followed by death."

Mr. *Field*, in treating this formidable disease, usually had recourse to excision of the wounded part (if any), and to subsequent cauterisation; also to venæsection, purgatives, and mercurial inunction of the whole body.* Many of the cases proved fatal. The morbid appearances, found on dissection, were—as in the human subject—by no means uniform or satisfactory. The lungs were usually very much congested, and the brain and spinal marrow in most instances highly injected, if not positively inflamed.

The following case of *Ligature* of the *Carotid Arteries*, which was successfully resorted to for the cure of a head-complaint, would have delighted the late *Caleb Parry* of Bath.

A horse had been long suffering for a cephalic affection—indicated by stupidity, occasional delirium, partial paralysis of the optic nerves, &c.—for which blisters and a depletory regimen had been fairly tried, but without success. The left carotid artery was accordingly exposed and tied. By the time the wound had healed, the animal had quite recovered from his disease. For nearly a twelvemonth afterwards, he remained well; but, then the cephalic symptoms returning, the other carotid was tied. In the course of a few days, the animal had quite recovered his senses in every respect; his appetite was good, and he was extremely lively and perfectly manageable.

*Hydrocephalus.*—A mare was brought to the hospital with inflammation of the brain. The active symptoms abated; but there remained great dulness of the eyes, and a *propensity to bore with the head.* A bleeding was ordered, and also purging medicine: the bleeding was repeated the same night. Next day the animal was so much better as to lead Mr. *Field* to form a favourable prognosis; but, on the following one, there was an aggravation of all the symptoms—great restlessness, much *boring*, stupor with occasional phrenzy. The animal was twice bled. Next day, in addition to the previous symptoms, the breathing was slow, the pulse intermittent and at times scarcely perceptible: at night she died. On *dissection*, the brain was found slightly inflamed; the two ventricles were distended with water; and two dark spots were found on each optic thalamus.

Mr. *Field* suggests that, as there was but little active inflammation of the brain in this case, blisters might probably have caused the absorption

* In one case, both carotid arteries were tied at once; but the animal died two hours afterwards. The experiment was—as admitted by the author—exceedingly injudicious. In another case alluded to, he tied them on two successive days; but without producing any decided effect, either beneficial or injurious to the patient.

of the water. *Query ?*—Was not the copious bleeding, so late in the disease, injurious ? We (*Rev.*) think it was ; and still more so in the next case recorded where the horse, after having exhibited certain signs of phrenitis, fell backwards. He was bled largely, purgatives given, and cold lotion applied to the head. At night he again fell down and was unable to rise ; the pulse was frequent, but without oppression. The *bleeding was repeated.* Next day he died ; and, on examining the brain, the lateral ventricles were found much distended with water, and in each a thick layer of lymph on the optic thalamus. (Had there been no lesion of vision during life ?).

Much mischief is often, we are sure, produced in the human subject, by having recourse to sanguineous depletion at an advanced stage of cerebral diseases ; and the same, we should suppose, holds true in the case of the horse. After having written this sentence, we accidentally turned to page 219, where there is a report of another case of Hydrocephalus, and were glad to find that our suspicion was confirmed by Mr. *Field's* own experience. The horse, as usual, had been freely bled, but rapidly became worse and died. On dissection, the substance of the brain was found quite pallid, and the lateral ventricles were filled with a transparent fluid. Our author very judiciously appends this remark :—

"The post-mortem examination in the above case, as well as the symptoms during life, forcibly and incontestably prove the fact, that, in proportion to the increasing quantity of serous fluid, so is there a diminution of blood in the brain and vessels ; and also, that an animal under such circumstances cannot bear the loss of blood—extreme restlessness, &c., almost immediately supervening." 219.

To illustrate still more clearly the character of Cephalic disease in the horse, we may here enumerate the symptoms observed in a case of *Encephalitis,* recorded by our author. They were—reeling about in the coach-house—boring against the wall—endeavouring to bite the groom—rushing at any one who approached him—squeaking and striking out when touched : the pulse was quiet. In leading him to the hospital, he nearly ran against some iron railings, in spite of two men who held him. On trying to put him into slings, he struggled so furiously that the attempt was given up. He would now paw, snort, and bore against the wall ; then he would rear up and get into the manger. He was freely bled, and for some time afterwards was more tranquil ; but again he became violent, reared, fell over, plunged and struggled furiously, and began to snort in the same suffocating manner, with retraction of the false nostrils. In this state he was laid on the ground, and then gradually sunk.

*Apoplexy* is another disease, to which horses are occasionally subject. Although the brain was not examined in the following case, we have no doubt as to the seat of the lesion.

A gelding, that seemed to be in good health, was put one afternoon into harness, as off-leader in a team. At starting he shewed a slight disposition to kick, and, by the motion of his head, appeared alarmed. Before he had gone many steps, he reeled against the near-leader ; otherwise he must have fallen. He was immediately bled to a large amount. The respiration was extremely laborious, with mucous rattle—pulse frequent and having a corded feel, but irregular and indistinct—tongue and lips livid—bleeding from both nostrils The pin was removed from the wound to allow more blood to flow; but he had not lost above a quart, when he knelt forwards and gradually fell on the ground; he died in about twenty minutes. (Here again, the loss of blood seems to have accelerated the death.)

The next case we select is one of *Apoplexy of the Spinal Cord.*

A horse, whilst exercising, fell suddenly on his head, and turned completely over. He lay almost without motion, and had not the slightest power to stand up. The vision was perfect, and the countenance natural. He tried to neigh several times, but could not give utterance to the sound. He did not struggle or express pain when lifted on, or removed from, the truck on which he was brought to the hospital. He could swallow, and passed some fæces. Death ensued in sixteen hours.

On dissection, the brain was found to be somewhat congested. On laying open the cervical portion of the spinal canal, it was found full of fluid, and a small quantity of blood was extravasated upon the *theca spinalis:* this must have occasioned pressure on the marrow.

*Purpura Hæmorrhagica* in the horse is characterised by the occurrence of petechial and ecchymosed spots and patches in almost every part and tissue of the body—on the skin, the Schneiderian membrane, the conjunctivæ of the eyes, under the fasciæ and in the substance of the muscles, and occasionally also in the lungs, kidneys, and other viscera. The animal is uneasy, and in pain; his breathing is usually hurried and snuffling; and his pulse is rapid and wiry, or full and strong. As the disease advances, the ecchymosed swellings become confluent; a sero-sanguineous discharge drips from the nostrils; the Schneiderian membrane is sometimes nearly black; the circulation is more frequent and feeble; *the horse paws, or looks back, or anxiously turns his head from side to side* (a graphic description); he totters, if made to walk about; becomes weaker, and dies. If blood be drawn, it is found to be thin and pallid, and separates rapidly into its two portions. The urine occasionally contains much albumen, and coagulates by nitric acid and heat: sometimes it is mixed with blood.

As Mr. Field remarks, the disease is seemingly referrible to a morbid condition—characterised chiefly by an increased tenuity—of the circulating fluids. There may indeed be present, at the same time, an unusual laxity and dilatability of the blood-vessels, with a certain degree of obstruction in the capillaries, and an augmented impetus in the heart's action; but certainly the primary and essential feature of Purpura is an alteration in the composition and vital properties of the blood itself. This opinion is confirmed by the circumstance of its usual exciting causes being general debility, poor living, unwholesome diet, and excessive labour.

As to the *treatment*, bleeding may be occasionally necessary, when vital organs are affected or threatened; "but it rarely (does it ever ?) benefits."*

---

* Mr. *Field* reports several cases, in which bleeding was practised: all these proved fatal. In some of the cases, the heart was found on dissection either paler than usual; or dark, flaccid and speckled. As a matter of course, these

Turpentine is useful; so are the sulphate of iron, the mineral acids, &c. The cold affusion is "particularly good." The pure air of the country, with grass feeding, is the best restorative.

In a fatal case of Hæmaturia, the peritoneal coat and cortical substance of the kidney (kidneys?) were entirely destroyed. The ureters were filled with blood: the bladder contained about two pints, mixed with urine. The liver was very pale; the intestines healthy. About a pint of blood was found within the pericardium. In another fatal case, where the quantity of blood lost had been enormous, the tubuli uriniferi and pelvis of the left kidney were found ulcerated, and the texture of the right one was soft and flabby.

We close our article with a few remarks on Calculus in the Bladder, and the history of a case of

*Lithotomy.*—The symptoms of calculus in the bladder are, as might be supposed, very much alike to those that are observed in the human subject. We read of the animal "staling blood after work,—continually making attempts to stale—straining violently and voiding only a small quantity at a time—and the urine being turbid and alkalescent." The actual presence of the stone is ascertained by an examination *per anum*, and by the introduction of a catheter or sound into the bladder. The following account of the mode, in which a horse is cut for the stone, is well worth a perusal: we cannot abridge it without injury.

"The animal was cast in the usual manner, and both hind legs were drawn to the shoulders, as if for castration. Read's new flexible catheter being passed into the bladder, a quantity of warm water was injected, sufficient to distend that organ and the urethra moderately. The catheter being withdrawn, and holding the penis with the left hand, a slightly-curved grooved staff, two feet long, was introduced, so as for the curved part to come into the sub-anal portion of the urethra, above the posterior edge of the ischium, extending towards the sphicter ani. An assistant, kneeling on the left side of the horse, drew the penis forwards with his left hand, and gently pushed the staff backwards with tbe right, at the same time keeping the groove exactly beneath the *raphe;* this elevated the portion of the urethra to be incised. I then made an incision, a line from and on the right side of the *raphe,* through the skin and fascia, extending the length of it from three to four inches; and, pushing the penis a little on one side, I gradually divided the muscular and spongy portion, and exposed the mucous membrane of the urethra, when the finger readily detected the groove of the staff, into which a small incision was made sufficient to admit the *bistoire cachée,* following which with the index finger of the left hand, the membrane was divided to the rectum. Very little blood flowed, and the water of the urethra gushed out. The staff being removed, I easily introduced the small forceps through the urethra into the bladder, and grasped the stone, a portion of which flaked off. The large forceps were then employed, and, my brother holding the handles, I directed the blades upon the stone, my left hand being in the rectum. Having placed the stone in a proper position, I grasped it with the forceps, and with both hands gave it a half-turn, so as to place its widest axis between the pubis and rectum; and thus, with a moderate force, I gradually and evenly drew it out,

---

are the signs not of a phlogistic, but of an adynamic, disease. We read, however, in the account of one dissection in a fatal case of Influenza; "Dreadful inflammation of the right side of the heart, which was almost black:"—was not this congestion, rather than inflammation?

the neck of the bladder readily dilating. Two stiches were inserted in that part of the incision nearest the anus, the lower part being left to itself." 117.

The calculus consisted entirely of carbonate of lime and animal matter. The animal did well.

A case is reported of Calculus in a mare. It was extracted, although with considerable difficulty, through the urethra by means of forceps, the bladder having been previously filled with warm water. It weighed upwards of 11 ozs. and measured $3\frac{3}{8}$ inches in length, and $2\frac{7}{8}$ in breadth.

Two ounces of laudanum were given immediately after the operation. Although rather unpleasant symptoms came on, the animal recovered perfectly.

A urinary Calculus, when small, is apt to become impacted in the urethra, and to give rise to retention of the water. Case 54 is an interesting example of this. An incision was made on the under surface of the urethra, and the stone—which was of the mulberry form, with projecting spiculæ on its surface—was extracted, to the great relief of the poor animal, which had been suffering excessively.

---

Lectures on the Principles and Practice of Physic, delivered at King's College, London. By *T. Watson*, M.D. London, 1843.

